# The effects of novel α_2_-adrenoreceptor agonist dexmedetomidine on shivering in patients underwent caesarean section

**DOI:** 10.1042/BSR20181847

**Published:** 2019-02-01

**Authors:** Gaofeng Yu, Shangyi Jin, Jinghui Chen, Weifeng Yao, Xingrong Song

**Affiliations:** 1 Department of Anesthesiology, Guangzhou Women and Children’s Medical Center, Guangzhou Medical University, Guangzhou, Guangdong, China; 2 Department of Vasculocardiology, the Third Affiliated Hospital of Sun Yat-sen University, Guangzhou, Guangdong, China; 3 Department of Anesthesiology, the Third Affiliated Hospital of Sun Yat-sen University, Guangzhou, Guangdong, China

**Keywords:** caesarean delivery, combined spinal-epidural anesthesia, dexmedetomidine, meperidine, shivering

## Abstract

**Objective:** Meperidine used to control shivering during perioperative period has associated side effects. The present study compared the safety of selective α2-adrenoreceptor agonist dexmedetomidine and meperidine for anti-shivering in primiparas after caesarean delivery under combined spinal-epidural anesthesia (CSEA).

**Methods:** 100 primiparas scheduled for caesarean delivery were randomly allocated to dexmedetomidine group (Group D, *n*=50) and meperidine positive control group (Group M, *n*=50). Primiparas experienced shivering that continued to cord clamping were treated with dexmedetomidine (0.5 μg/kg) or meperidine (0.5 mg/kg) after cord clamping. The primary outcome measures were incidence of nausea, vomiting, and respiratory depression. Secondary outcome measures were shivering score, vital signs including blood pressure, heart rate and O_2_ saturation, tympanic temperature, and sedation score.

**Results:** Dexmedetomidine provided similar anti-shivering effects as meperidine in patients after caesarean delivery under CSEA, evidenced as all shivering primiparas responded to either dexmedetomidine or meperidine treatment within 15 min. However, incidence of nausea and vomiting were significantly lower after dexmedetomidine treatment, accompanied with more stable blood pressure. Dexmedetomidine also provided well regulation of tympanic temperature and good sedation.

**Conclusion:** Selective α_2_-adrenoreceptor agonist dexmedetomidine has a better safety profile compared with meperidine for anti-shivering in primiparas undergoing caesarean delivery. Dexmedetomidine could be a better choice for anti-shivering in patients requiring caesarean section. The mechanism of anti-shivering for dexmedetomidine may relate to well regulation of temperature and good sedation.

## Introduction

Shivering is common during cesarean section under spinal anesthesia and may continue for several hours after delivery. Shivering during delivery under spinal anesthesia is uncomfortable for patients and can increase oxygen consumption, carbon dioxide production, and lactic acid accumulation. Furthermore, patient shivering may interfere with blood pressure and oxygen saturation monitoring as well as electrocardiograms. The redistribution of core temperature contributes to shivering under spinal anesthesia [1]. Compared with other patients, large body surface area of maternal gives rise to greater heat loss after combined spinal-epidural anesthesia (CSEA) during cesarean section. In addition, the unique process of amniotic fluid loss in cesarean section, should certainly accompanied by extra heat loss. Therefore, the incidence of shivering in parturient undergoing cesarean section with CSEA is higher. It was reported that the incidence of shivering in cesarean section with regional anesthesia was higher than that in other procedures (up to 55%) [2]. In addition, the rate of caesarean sections is increasing yearly [3,4]. More and more parturient will struggle with shivering during cesarean section with CSEA.

Opioids, α_2_-agonists, and 5-hydroxytrypatamine agonists or antagonists have been used clinically to control shivering in the perioperative period [5]. Among these, meperidine is the medication most often recommended for clinical use. Meperidine’s anti-shivering effects have been attributed to its actions on the κ-opioid receptor [6] and the α_2b_ adrenoreceptor subtype [7]. Side effects associated with meperidine administration include nausea, vomiting, and pruritus [8], as well as respiratory depression at higher doses [9].

Dexmedetomidine is a selective α_2_-adrenoreceptor agonist that causes sedation, anxiety reduction, and analgesia without the development of respiratory depression [10]. Some evidence [11] suggests that dexmedetomidine can reduce post anesthetic shivering after surgery. Dexmedetomidine has been used previously to prevent postoperative shivering in patients undergoing elective total abdominal hysterectomy under general anesthesia (fentanyl, propofol, atracurium) [12].

As clinical experience suggests that dexmedetomidine may be effective in reducing the incidence of shivering in patients during and after caesarean section under spinal anesthesia, we hypothesized that dexmedetomidine has better efficacy for anti-shivering in this patient population, with less side effects than meperidine. This prospective, double-blind, randomized clinical study compared the efficacy and safety of dexmedetomidine and meperidine for reducing the incidence and severity of shivering in primiparas after caesarean delivery under CSEA.

## Materials and methods

This trial was registered at http://www.chictr.org.cn/index.aspx (ChiCTR-IPR-15005860; Principal investigators: Gaofeng Yu, Jinghui Chen, Xingrong Song) on January 21, 2015. The trial was approved by the human investigation committee of Guangzhou Women and Children’s Medical Center and was conducted in accordance with the principles of the World Medical Association Declaration of Helsinki [13] and Good Clinical Practice guidelines [14].

### Patients

Healthy primiparas scheduled for caesarean delivery under CSEA were eligible for the present study. Inclusion criteria were (1) American Society of Anesthesiologists (ASA) physical status I or II; (2) age ≥ 18 years; and (3) shivering during cesarean delivery that continued to cord clamping. Exclusion criteria were (1) febrile illness; (2) hypertension; (3) diabetes mellitus; (4) abnormal liver or renal function; or (5) coagulation disorders.

Using a computer-generated random code and the sealed-envelope system, included primiparas were randomly allocated 1:1 to a dexmedetomidine group (Group D, *n*=50) or a meperidine group (Group M, *n*=50). All subjects provided written informed consent. Primiparas and the anesthetist were unaware of the study group allocations. A nurse anesthetist that was not involved in data recording or analysis prepared the medications and marked them “A” or “B”.

### Study procedures

The operating room was maintained at 24–26°C and 60% humidity. CSEA was performed at L3-4 or L2-3 interspace after infiltration of 2 ml 0.5% ropivocaine for spinal anesthesia and 3 ml 1% lidacaine as the epidural test dose. Patients breathed air and were covered in sheets; no active heating method was adopted during anesthesia. Fluids and medications were administered at room temperature. If primiparas shivered before cord clamping, dexmedetomidine 0.5 µg/kg (diluted with normal saline to 20 ml) or meperidine 0.5 mg/kg (diluted with normal saline to 20 ml) was administered intravenously by pump (120 ml/h, 10 min). If shivering lasted more than 15 min, the treatment was considered invalid. If treatments were not effective, 4 mg ondansetron could be administered intravenously as a rescue medicine; if it was necessary, 1 mg/kg ketamine administered intravenously could be adopted.

### Sample size

The primary outcome measure was the incidence of nausea. A pilot study in our group found that the incidence of nausea was 50% and 10% for the meperidine group and the dexmedetomidine group, respectively. With a power of 90% and a type I error of 5%, 42 patients per group were required to achieve acceptable results. Accounting for 20% of dropouts, 50 patients were recruited to each study group.

### End points

The primary outcome measure was incidence of adverse events, including nausea, vomiting, and respiratory depression. Secondary outcome measures were shivering score, vital signs including systolic blood pressure (SBP), diastolic blood pressure (DBP), mean blood pressure (Pmean), heart rate (HR), O_2_ saturation (SpO_2_), tympanic temperature, and sedation score.

Duration of surgery, infusion volume, amount of bleeding, urine volume, the highest plane of sensory block, minimum HR, dose of atropine administered for bradycardia, adverse events including nausea, vomiting, respiratory depression, bradycardia, and Apgar scores of the newborns at 1, 5, and 10 min were recorded.

Shivering score was measured when shivering appeared. Shivering was graded according to a validated scale [15] where 0 = no visible shivering; 1 = no visible shivering, but peripheral vasoconstriction or piloerection was observed; 2 = activity in one muscle group only; 3 = activity in more than one muscle group, but shivering was not generalized; 4 = shivering involving the whole body.

Sedation was graded according to the Ramsey sedation scale: 1 = patient awake, anxious, agitated, restless; 2 = patient awake, cooperative, oriented, tranquil; 3 = patient awake, responsive to commands only; 4 = patient asleep, brisk response to light glabellar tap or loud auditory stimulus; 5 = patient asleep, sluggish response to light glabellar tap or loud auditory stimulus; 6 = patient asleep, no response to light glabellar tap or loud auditory stimulus.

Vital signs, tympanic temperature monitored with an ear thermometer (Thermoscan IRT 3020; Braun, Kronberg, Germany), and the sedation score were recorded at the following time points: before (Tb) and after (Ta) CSEA, at cord clamping (Tc), and at 0 min (T0), 5 min (T5), 10 min (T10), 15 min (T15), 20 min (T20), and 30 min (T30) after administration of dexmedetomidine (Jiangsu Hengrui Medicine Co., Ltd.) or meperidine (Hubei Yichang Renfu Pharmaceutical Co., Ltd).

### Statistical analysis

Statistical analysis was conducted using SPSS software version 16.0 (IBM, Armonk, NY, U.S.A.). Quantitative variables including shivering score, primipara age, gestational age, weight, height, body mass index (BMI), duration of surgery, infusion quantity, amount of bleeding, urine volume, the highest plane of sensory block, minimum HR, and dose of atropine were compared between groups using the Mann–Whitney *U*-test or the Kruskal–Wallis test. Sedation score, baseline and intraoperative vital signs (HR, SBP, DBP, Pmean, and SpO_2_), tympanic temperature, and Apgar score were evaluated with repeated-measures analysis of variance (ANOVA). Data are expressed as mean ± SD; *P*<0.05 was considered statistically significant.

## Results

### Basic information of patient

From February 2, 2015 to September 28, 2015, a total of 100 primiparas underwent randomization: 50 to the dexmedetomidine group (Group D) and 50 to the meperidine group (Group M). Morphometric characteristics (primipara age, gestational age, weight, height, BMI) and duration of surgery were similar between the two groups. The highest plane of sensory block, infusion volume, amount of bleeding, urine volume, minimum HR, and the Apgar score at 1, 5, and 10 min did not differ between groups. Atropine was not administered to any patient (Table 1).

**Table 1:** Characteristics of patients in two groups

Parameter	Group D (*n=33*)	Group M (*n=35*)	*P* value
Age (years)	31.24 ± 3.95	31.54 ± 3.15	0.73
Gestational age (weaks)	38.9 ± 0.88	38.48 ± 1.07	0.08
Weight (kg)	73.42 ± 4.71	74.21 ± 3.56	0.44
Height (m)	1.58 ± 0.04	1.58 ± 0.04	0.78
BMI	29.38 ± 2.12	29.60 ± 1.82	0.64
Highest plane of sensory block	6.06 ± 0.90	6.03 ± 0.62	0.86
Duration of surgery (min)	53.58 ± 5.14	54.83 ± 6.42	0.38
Infusion quantity (ml)	845.15 ± 59.06	873.43 ± 57.95	0.05
Amount of bleeding (ml)	278.79 ± 33.89	276.00 ± 30.21	0.72
Urine volume (ml)	193.94 ± 57.98	209.14 ± 50.20	0.25
The lowest HR (bpm)	68.61 ± 6.13	68.26 ± 5.47	0.81
Apgar 1 min	9.36 ± 0.55	9.46 ± 0.51	0.47
Apgar 5 min	9.48 ± 0.51	9.46 ± 0.51	0.82
Apgar 10 min	9.88 ± 0.33	9.86 ±0.36	0.80
Shivering score	2.58 ± 0.75	2.51 ± 0.70	0.73
Dosage of atropine (mg)	0	0	

Data are mean ± SD.

### Selective α_2_-adrenoreceptor agonist dexmedetomidine effectively reduced post operation shivering

**Figure 1:**
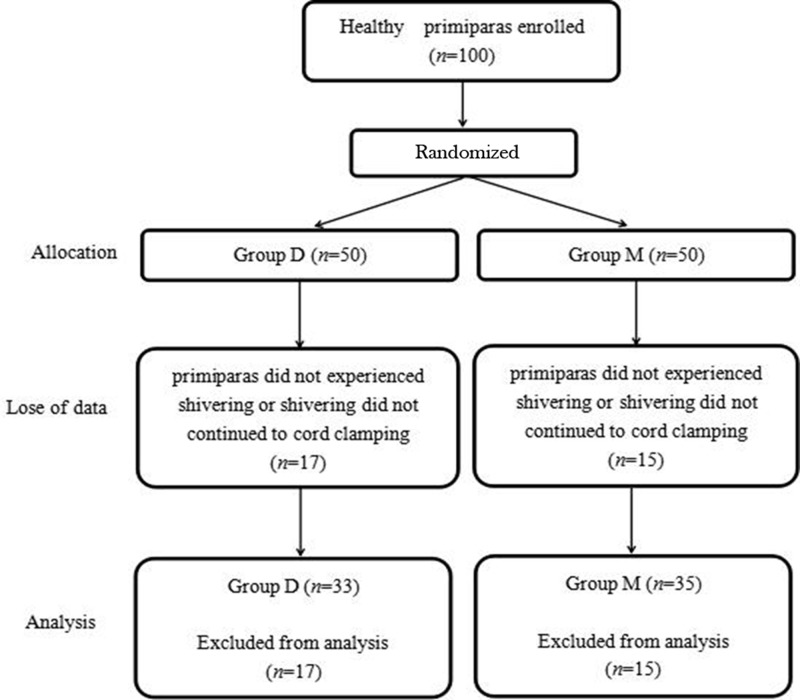
Flow chart of patients

As shown in Figure 1, all shivering primiparas responded to either dexmedetomidine or meperidine treatment within 15 min. A total of 33 primiparas in dexmedetomidine treatment group and 35 primiparas in meperidine treatment group experienced shivering (Group D: 66.00%, 33/50 vs. Group M: 70.00%, 35/50; *P*=0.67). However, except the well anti-shivering effects of dexmedetomidine, the incidence of nausea and vomiting were significantly lower in dexmedetomidine treatment group compared with meperidine treatment group (*P*<0.01) (Table 2). These results indicating that selective α2-adrenoreceptor agonist dexmedetomidine existed anti-shivering effects with less adverse reactions (nausea and vomiting) in patients underwent caesarean section.

**Table 2:** Incidence of nausea and vomiting

	Group D	Group M	*P*
	*n*=33	*n*=35	
Nausea	1/33, 3%	18/35, 51.4%	<0.01
Vomiting	0/33, 0%	7/35, 20%	0.01

### Dexmedetomidine improved circulatory stability

**Figure 2:**
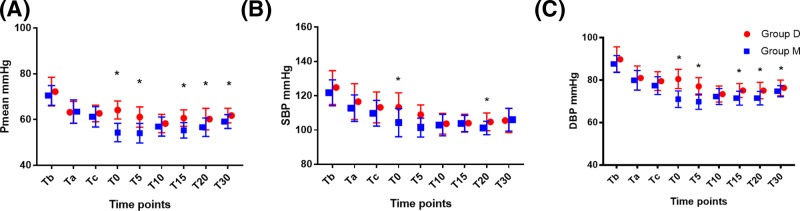
Change in blood pressure during the study period Blood pressure was monitored at the following time points: before (Tb) and after (Ta) CSEA, cord clamping (Tc), 0 min (T0), 5 min (T5), 10 min (T10), 15 min (T15), 20 min (T20), and 30 min (T30) after administration of dexmedetomidine or meperidine. (**A**) Pmean, (**B**) SBP, (**C**) DBP. *Compared with Group M, *P*<0.05. Group D means patients received dexmedetomidine treatment during caesarean section. Group M means patients received meperidine treatment during caesarean section.

After anesthesia, blood pressure decreased slightly in both groups, but no ephedrine was needed to treat potential hypotension. Blood pressure plateaued after T5 in both groups. Pmean was significantly higher at T0, T5, T15, T20, and T30 (Figure 2A), SBP was significantly higher at T0 and T20 (Figure 2B), and DBP was significantly higher at T0, T5, T15, T20, and T30 (Figure 2C) in dexmedetomidine treatment group compared with meperidine treatment group. These findings suggest blood pressure was more stable in dexmedetomidine treatment group compared with meperidine treatment group.

**Figure 3:**
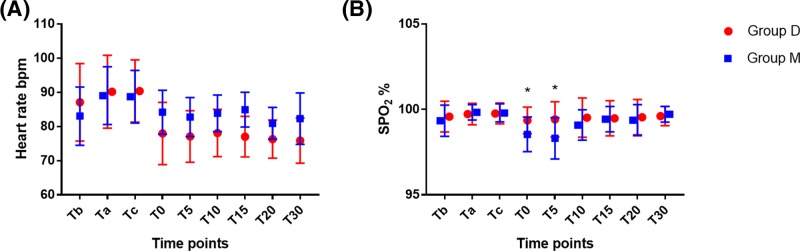
Change in HR and SpO_2_ during the study period HR and SpO_2_ were monitored at the following time points: before (Tb) and after (Ta) CSEA, cord clamping (Tc), 0 min (T0), 5 min (T5), 10 min (T10), 15 min (T15), 20 min (T20), and 30 min (T30) after administration of dexmedetomidine or meperidine. (**A**) HR and (**B**) O_2_ saturation (SpO_2_). *Compared with Group M, *P*<0.05. Group D means patients received dexmedetomidine treatment during caesarean section. Group M means patients received meperidine treatment during caesarean section.

Following dexmedetomidine or meperidine administration, there was no significant decrease in HR. HR was higher in meperidine treatment group compared with dexmedetomidine treatment group at all time points after treatment, but the differences were not significant (Figure 3A). SpO_2_ was significantly higher at T0 and T5 in dexmedetomidine treatment group compared with meperidine treatment group (Figure 3B).

### Dexmedetomidine provided well regulation of temperature and good sedation

**Figure 4:**
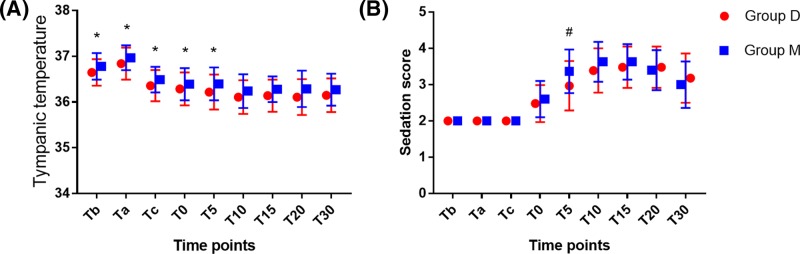
Change in tympanic temperature during the study period Tympanic temperature was monitored at the following time points: before (Tb) and after (Ta) CSEA, cord clamping (Tc), 0 min (T0), 5 min (T5), 10 min (T10), 15 min (T15), 20 min (T20), and 30 min (T30) after administration of dexmedetomidine or meperidine. (**A**) Patients tympanic temperature. (**B**) Patients sedation score. *Compared with T10, *P*<0.05; ^#^compared with Group M, *P*<0.05. Group D means patients received dexmedetomidine treatment during caesarean section. Group M means patients received meperidine treatment during caesarean section.

Following dexmedetomidine or meperidine administration, there was a significant decrease in tympanic temperature from Tc to T0–30 (Table 3). Tympanic temperature plateaued 10 min after administration of dexmedetomidine or meperidine (Figure 4A). There was no significant difference in tympanic temperature between groups at any timepoint (*F* = 3.27, *P*=0.08). These findings suggest that dexmedetomidine and meperidine had similar effects on the thermoregulatory center.

**Table 3:** Tympanic temperature (^o^C)

Group	Time points								
	Tb	Ta	Tc	T0	T5	T10	T15	T20	T30
Group D (*n*=33)	36.65 ± 0.29	36.84 ± 0.35	36.36 ± 0.34	36.29 ± 0.36^*a*^	36.22 ± 0.38^*a*^	36.11 ± 0.37^*a*^	36.14 ± 0.35^*a*^	36.11 ± 0.41^*a*^	36.15 ± 0.37^*a*^
Group M (*n*=35)	36.78 ± 0.29	36.97 ± 0.27	36.49 ± 0.28	36.39 ± 0.35^*b*^	36.40 ± 0.36^*b*^	36.24 ± 0.37^*b*^	36.28 ± 0.28^*b*^	36.29 ± 0.40^*b*^	36.27 ± 0.35^*b*^

Data are mean ± SD. Tympanic temperature was monitored at the following time points: before (Tb) and after (Ta) CSEA, cord clamping (Tc), 0 min (T0), 5 min (T5), 10 min (T10), 15 min (T15), 20 min (T20), and 30 min (T30) after administration of dexmedetomidine or meperidine.

^a^Compared with Tc in Group D, *P*<0.05.

^b^Compared with Tc in Group M, *P*<0.05.

Sedation status affects the incidence of shivering. Sedation score increased but then plateaued 5 min after treatment with dexmedetomidine or meperidine. Sedation score was significantly lower at T5 in dexmedetomidine treatment group (2.97 ± 0.68 compared with meperidine treatment group (3.37 ± 0.60) (*P*=0.01) (Figure 4B). These results suggested that the mechanism of anti-shivering for dexmedetomidine may relate to well regulation of temperature and good sedation.

## Discussion

The present study investigated the efficacy and safety of dexmedetomidine (0.5 µg/kg) and meperidine (0.5 mg/kg) for reducing the incidence and severity of shivering in primiparas after caesarean delivery under CSEA. Mean incidence of shivering before treatment was 68% (Group D, 66%, 33/50; Group M, 70%, 35/50), which was similar to the findings from other studies [16,17]. All shivering primiparas responded favorably to dexmedetomidine or meperidine treatment, both of which stopped primiparas’ shivering within 15 min of administration.

The exact mechanism of shivering in primiparas under CSEA has not been fully established, but shivering may be caused by loss of heat to the environment, inhibition of central thermoregulatory control, and redistribution of body heat [18,19]. In the clinical setting, shivering is most often prevented or treated using pharmacological management. Meperidine is generally recommended for the control of postanesthetic shivering. The mechanism and site of action of meperidine as an anti-shivering agent have yet to be fully elucidated. Evidence suggests that the anti-shivering effects of meperidine can be minimally blocked by low-dose naloxone (blocking most μ-receptors), and totally reversed by high-dose naloxone (blocking all μ- and most κ-receptors) [20]. Accordingly, meperidine may exert its anti-shivering effects, at least in part, on κ-opioid receptors. However, meperidine may also act directly on the thermoregulatory center [21].

Dexmedetomidine is a centrally acting a_2_-adrenergic agonist with a short half-life (2–3 h vs. 12–24 h for clonidine, another classic α_2_-adrenergic agonist). Because of its potentially beneficial physiologic effects, dexmedetomidine is widely used for sedation in mechanical ventilation, procedural sedation, prevention of acute agitation, and neuroprotection [22–27]. Dexmedetomidine may exert its anti-shivering effects by acting on the central thermoregulatory system and reducing vasoconstriction and the shivering threshold [28]. We had the similar result that the high dose of dexmedetomidine (0.5 µg/kg) effectively controlled shivering compared with the study of Abdel-Ghaffar et al. (100% vs. 96.7%) [28]. The deficiencies related to used dose was hemodynamic instability and excessive sedation, that may be related to venous bolus injection (>2 min) despite the changes of blood pressure and HR rate tend to be reversible over time. Given that, then we accepted a modified approach (constant infusion for 10 min) in the present study, the development of hemodynamic within 30 min suggested that systolic pressure was more stable even though had not fully eliminated the common complications of bradycardia and sedation. Determining the exact dose of dexmedetomidine 0.3 µg/kg or 0.5 µg/kg in our or any other study is notoriously difficult balanced efficiency and time. Multiple factors may contribute to dosing strategies used for shivering including preexisting patient condition, types of surgery, duration of operation, as well as personal experience. Finally, the high dose of dexmedetomidine (0.5 µg/kg) used in the present study with constant infusion for 10 min had displayed positive effects without serious adverse event.

It is well known that opioid-induced nausea, vomiting, and respiratory depression result from activation of the μ-receptor. Dexmedetomidine is a highly selective α_2_-adrenergic agonist that has no affinity for the μ-receptor, while meperidine is a classic agonist of the μ-receptor. In the present study, there was a lower incidence of nausea and vomiting in dexmedetomidine treatment group compared with meperidine treatment group (nausea, Group D vs. Group M = 3.03% vs. 51.43%, *P*<0.01; vomiting, Group D vs. Group M = 0.00% vs. 20.00%, *P*=0.01). Respiratory depression was not observed in ether group, presumably due to the low doses of medications used and the slow rates of infusion. Following dexmedetomidine or meperidine administration SpO_2_ decreased slightly, and the decrease in SpO_2_ was less in dexmedetomidine treatment group compared with meperidine treatment group. Blood pressure was more stable in dexmedetomidine treatment group compared with meperidine treatment group. Dexmedetomidine-induced bradycardia was one concern, but mean minimum HR was not significantly different between the two groups; no atropine was administered to any patient during the study period.

One limitation of the present study was that we just evaluated the effects of dexmedetomidine and meperidine on newborns by Apgar Scores. Umbilical arterial pH reflected situation of newborns and was related to shivering of patients, too. It was another valuable indicator which could be involved in our further study.

**Figure 5:**
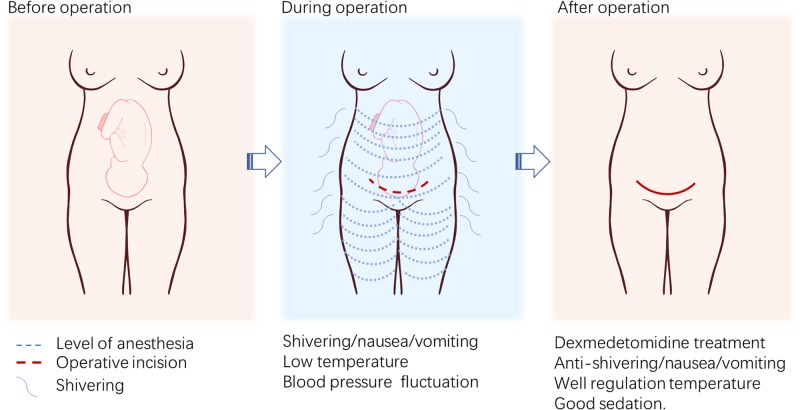
Schematic diagram of selective α_2_-adrenoreceptor agonist dexmedetomidine for anti-shivering in primiparas undergoing caesarean delivery under CSEA Dexmedetomidine provided anti-shivering effects in patients after caesarean delivery under CSEA. Moreover, incidence of nausea and vomiting were significantly lower after dexmedetomidine treatment, accompanied with more stable blood pressure. Dexmedetomidine also provided well regulation of tympanic temperature and good sedation.

In conclusion, data from the present study indicate that dexmedetomidine (0.5 µg/kg) has similar efficacy and a better safety profile compared with meperidine (0.5 mg/kg) for the control of shivering in primiparas undergoing cesarean section under CSEA. Dexmedetomidine could be a better choice for the control of shivering in patients requiring caesarean section under CSEA (Figure 5).

## Abbreviations

BMI: body mass index
CSEA: combined spinal-epidural anesthesia
DBP: diastolic blood pressure
HR: heart rate
Pmean: mean blood pressure
SBP: systolic blood pressure

## References

[1] Kurz A., Sessler D.I., Schroeder M. and Kurz M. (1993) Thermoregulatory response threshold during spinal anesthesia. Anesth. Analg. 77, 721–726 10.1213/00000539-199310000-00011 8214655

[2] Hong J.Y. and Lee I.H. (2005) Comparison of the effects of intrathecal morphine and pethidine on shivering after caesarean delivery under combined-spinal epidural anaesthesia. Anaesthesia 60, 1168–1172 10.1111/j.1365-2044.2005.04158.x 16288613

[3] Hassani L., Aghamolaei T. and Ghanbarnejad A. (2016) The effect of an instructional program based on health belief model in decreasing cesarean rate among primiparous pregnant mothers. J. Educ. Health Promot. 5, 1–1 10.4103/2277-9531.184558 27512693 PMC4959261

[4] Kozhimannil K.B., Law M.R. and Virnig B.A. (2013) Cesarean delivery rates vary tenfold among US hospitals; reducing variation may address quality and cost issues. Health aff. (Millwood) 32, 527–535 10.1377/hlthaff.2012.1030 23459732 PMC3615450

[5] Mahmood M.A. and Zweifler R.M. (2007) Progress in shivering control. J. Neurol. Sci. 261, 47–54 10.1016/j.jns.2007.04.038 17512551

[6] Solhpour A., Jafari A., Hashemi M. (2016) A comparison of prophylactic use of meperidine, meperidine plus dexamethasone, and ketamine plus midazolam for preventing of shivering during spinal anesthesia: a randomized, double-blind, placebo-controlled study. J. Clin. Anesth. 34, 128–135 10.1016/j.jclinane.2016.03.036 27687359

[7] Takada K., Clark D.J., Davies M.F. (2002) Meperidine exerts agonist activity at the α_2b_-adrenoreceptor subtype. Anesthesiology 96, 1420–1426 10.1097/00000542-200206000-00022 12170055

[8] Anaraki A.N. and Mirzaei K. (2012) The effect of different intrathecal doses of meperidine on shivering during delivery under spinal anesthesia. Int. J. Prev. Med. 3, 706–712 23112897 PMC3482998

[9] Ong B. and Segstro R. (1994) Respiratory depression associated with meperidine spinal anaesthesia. Can. J. Anaesth. 41, 725–727 10.1007/BF03015628 7923521

[10] Vaughns J.D., Martin C., Nelson J., Nadler E. and Quezado Z.M. (2017) Dexmedetomidine as an adjuvant for perioperative pain management in adolescents undergoing bariatric surgery: an observational cohort study. J. Pediatr. Surg. 52, 1787–1790 10.1016/j.jpedsurg.2017.04.007 28465076

[11] Bicer C., Esmaoglu A., Akin A. and Boyaci A. (2006) Dexmedetomidine and meperidine prevent postanaesthetic shivering. Eur. J. Anaesthesiol. 23, 149–153 10.1017/S0265021505002061 16426470

[12] Elvan E. G., Öç B., Uzun Ş., Karabulut E., Coşkun F. and Aypar Ü. (2008) Dexmedetomidine and postoperative shivering in patients undergoing elective abdominal hysterectomy. Eur. J. Anaesthesiol. 25, 357–364 10.1017/S0265021507003110 18205960

[13] World Medical Association Declaration of Helsinki (2000) Ethical principles for medical research involving human subjects. J. Am. Med. Assoc. 284, 3043–3045 10.1001/jama.284.23.3043 11122593

[14] International Conference on Harmonisation Expert Working Group (2001) ICH Harmonised Tripartite Guideline: Guideline for Good Clinical Practice. J Postgrad Med. 47, 199–203 11832625

[15] Sankar-Maharaj S., Chen D. and Hariharan S. (2018) Postoperative shivering among Cannabis users at a public hospital in Trinidad, West Indies. J. Perianesth. Nurs. 33, 37–44 10.1016/j.jopan.2016.03.010 29362045

[16] Chow T.C. and Cho P.H. (1994) The influence of small dose intrathecal fentanyl on shivering during transurethral resection of prostate under spinal anesthesia. Acta Anaesthesiol. Sin. 32, 165–170 7921861

[17] Kararmaz A., Kaya S., Turhanoglu S. and Ozyilmaz M.A. (2003) Low-dose bupivacaine-fentanyl spinal anaesthesia for transurethral prostatectomy. Anaesthesia 58, 526–530 10.1046/j.1365-2044.2003.03153.x 12846615

[18] Kishore N., Payal Y.S., Kumar N. and Chauhan N. (2016) In spinal anaesthesia for cesarean section the temperature of bupivacaine affects the onset of shivering but not the incidence: a randomized control trial. J. Clin. Diagn. Res. 10, UC18–UC21 26894154 10.7860/JCDR/2016/15883.7118 PMC4740682

[19] Yu S., Qualls-Creekmore E., Rezai-Zadeh K. (2016) Glutamatergic preoptic area neurons that express leptin receptors drive temperature-dependent body weight homeostasis. J. Neurosci. 36, 5034–5046 10.1523/JNEUROSCI.0213-16.2016 27147656 PMC4854966

[20] Zhou C., Zhu Y., Liu Z. and Ruan L. (2016) 5-HT3 receptor antagonists for the prevention of postoperative shivering: a meta-analysis. J. Int. Med. Res. 44, 1174–1181 10.1177/0300060516668776 27856931 PMC5536761

[21] Luggya T.S., Kabuye R.N., Mijumbi C., Tindimwebwa J.B. and Kintu A. (2016) Prevalence, associated factors and treatment of post spinal shivering in a Sub-Saharan tertiary hospital: a prospective observational study. BMC Anesthesiol. 16, 100 10.1186/s12871-016-0268-0 27756210 PMC5070127

[22] Di M., Yang Z., Qi D. (2018) Intravenous dexmedetomidine pre-medication reduces the required minimum alveolar concentration of sevoflurane for smooth tracheal extubation in anesthetized children: a randomized clinical trial. BMC Anesthesiol. 18, 9 10.1186/s12871-018-0469-9 29343232 PMC5773144

[23] Shutes B.L., Gee S.W., Sargel C.L., Fink K.A. and Tobias J.D. (2018) Dexmedetomidine as single continuous sedative during noninvasive ventilation: typical usage, hemodynamic effects, and withdrawal. Pediatr. Crit. Care Med. 19, 287–297 10.1097/PCC.0000000000001451 29341985

[24] Chen J., Li H., Lim G. (2018) Different effects of dexmedetomidine and midazolam on the expression of NR2B and GABAA-α1 following peripheral nerve injury in rats. IUBMB Life 70, 143–152 10.1002/iub.1713 29341457

[25] Prielipp R.C., Wall M.H., Tobin J.R. (2002) Dexmedetomidine-induced sedation in volunteers decreases regional and global cerebral blood flow. Anesth. Analg. 95, 1052–1059 12351293 10.1097/00000539-200210000-00048

[26] Han Y., Han L., Dong M. (2018) Comparison of a loading dose of dexmedetomidine combined with propofol or sevoflurane for hemodynamic changes during anesthesia maintenance: a prospective, randomized, double-blind, controlled clinical trial. BMC Anesthesiol. 18, 12 10.1186/s12871-018-0468-x 29368594 PMC5784722

[27] Yeh C.H., Hsieh L.P., Lin M.C. (2018) Dexmedetomidine reduces lipopolysaccharide induced neuroinflammation, sickness behavior, and anhedonia. PLoS One 13, e0191070 10.1371/journal.pone.0191070 29351316 PMC5774758

[28] Abdel-Ghaffar H.S., Mohamed S.A., Fares K.M. and Osman M.A. (2016) Safety and efficacy of dexmedetomidine in treating post spinal anesthesia shivering: a randomized clinically controlled dose-finding trial. Pain Physician 19, 243–253 27228512

